# In Ovarian Cancer Multicellular Spheroids, Platelet Releasate Promotes Growth, Expansion of ALDH+ and CD133+ Cancer Stem Cells, and Protection against the Cytotoxic Effects of Cisplatin, Carboplatin and Paclitaxel

**DOI:** 10.3390/ijms22063019

**Published:** 2021-03-16

**Authors:** Naike Casagrande, Cinzia Borghese, Francesco Agostini, Cristina Durante, Mario Mazzucato, Alfonso Colombatti, Donatella Aldinucci

**Affiliations:** 1 Molecular Oncology Unit, Centro di Riferimento Oncologico di Aviano (CRO), IRCCS, 33081 Aviano, Italy; naike.casagrande@cro.it (N.C.); cpborghese@cro.it (C.B.); acolombatti@cro.it (A.C.); 2 Stem Cell Unit, Centro di Riferimento Oncologico di Aviano (CRO) IRCCS, 33081 Aviano, Italy; fagostini@cro.it (F.A.); cdurante@cro.it (C.D.); mmazzucato@cro.it (M.M.)

**Keywords:** platelet releasate, ovarian cancer stem cells, ovarian cancer spheroids, anticancer therapy, drug resistance, cisplatin, carboplatin, paclitaxel

## Abstract

A high platelet count is associated with a poor prognosis in ovarian cancer (OvCa). Despite good clinical responses with platinating agents in combination with taxanes, numerous OvCa patients relapse due to chemotherapy resistance. Here, we report that treatment of OvCa cells A2780, OVCAR5 and MDAH with releasate from activated platelets (PR) promoted multicellular tumor spheroid (MCTS) formation. These OvCa-MCTSs had increased percentages of CD133+ and aldehyde dehydrogenase (ALDH)+ cells, bona fide markers of OvCa cancer stem cells (CSCs). PR increased OVCAR5- and MDAH-MCTS viability and decreased the cytotoxic and pro-apoptotic effects of paclitaxel, cisplatin and carboplatin. PR increased the volume of spontaneously formed OVCAR8-MCTSs and counteracted their size reduction due to cisplatin, carboplatin and paclitaxel treatment. PR promoted the survival of ALDH+ and CD133+ OvCa cells during cisplatin, carboplatin and paclitaxel treatment. In conclusion, molecules and growth factors released by activated platelets (EGF, PDGF, TGF-β, IGF and CCL5) may protect tumor cells from chemotherapy by promoting the expansion of ALDH+ and CD133+ OvCa-CSCs, favoring drug resistance and tumor relapse.

## 1. Introduction

An increased platelet count is correlated with poor disease-free survival (DFS) and overall survival in various cancers [1,2,3], including ovarian cancer (OvCa) [4,5,6,7]. Indeed, platelets and molecules secreted by platelets affect tumor growth, metastasis and anticancer drug activity [8,9,10,11,12]. In turn, tumor cells can stimulate platelet production and activation, leading to an abnormally increased number of platelets in the blood (thrombocytosis) and to the release of growth factors, cytokines, chemokines and vesicular signals, collectively termed “platelet releasate” (PR) [12,13,14]. PR contains vascular endothelial growth factor (VEGF) and fibroblast growth factor (FGF), which stimulate angiogenesis; TGF-beta, which promotes tumor growth; and CCL5, which is involved in cancer progression and cisplatin resistance [15,16,17]. Preclinical and clinical studies suggest the involvement of platelets and their products in OvCa progression [14,15,18,19]. In patients with OvCa, paraneoplastic trombocytosis [4] and a high platelet count were negatively correlated with the response to taxane-based therapies [5,7] and abundant platelets have also been found in tumor tissues and in ascitic fluids [6,20]. The co-culture of platelets with OvCa cells increased tridimensional multicellular tumor spheroid (MCTS) formation, with the expression of metastasis-initiating cell markers and migration [20], which was inhibited by aspirin [21]. PR increased the proliferation and protected OvCa cells from apoptosis (in a two-dimensional model) through a mechanism that did not require a direct cell–cell contact [5,22]. Similarly, PR increased tumor growth and decreased docetaxel activity in OvCa xenograft models [5].

Considering this evidence, our hypothesis was that growth factors secreted by platelets, by promoting the expansion of the putative drug-resistant CD133+ and ALDH+ OvCa cancer stem cells (CSCs), could decrease the anticancer activity of paclitaxel (PTX), cisplatin (CDDP) and carboplatin (CBDCA), the standard postoperative chemotherapy medications [23] for OvCa, and could thus justify the poor prognosis observed in patients with high platelet counts [11].

Using drug-resistant three-dimensional multicellular OvCa MCTSs [24,25,26], we studied the effects of molecules released by physiological activated platelets (PR) [27,28,29] on OvCa-CSC expansion and PTX, CDDP and CBDCA treatments.

Here, we show that PR increased OvCa-MCTS formation, as well as growth and the expression of the CSCs markers ALDH and CD133, whereas it decreased CDDP, CBDCA and PTX cytotoxicity, maintaining high levels of CSC markers.

## 2. Results and Discussion

OvCa patients are responsive to chemotherapy (cisplatin, carboplatin and docetaxel) at first; however, the main obstacles for successful chemotherapy are represented by drug toxicity and drug resistance [23]. Drug resistance can be intrinsic, acquired or achieved by the aggregation of tumor cells as spheroids or heterospheroids (the interaction of tumor cells with normal cells). These aggregates of tumor cells, called multicellular tumor spheroids (MCTSs) are less sensitive to anticancer drugs and are capable of disseminating in the peritoneum to form metastasis [30,31,32], and are found in malignant ascites formed in late-stage OvCa patients with peritoneal carcinomatosis. Subpopulations of these tumor cells exhibit CSC features. They self-renew, grow as MCTSs, express CD133 and ALDH, and possess enhanced resistance to chemotherapy [33,34,35]. Malignant ascites can contain platelets, free or associated with OvCa-MCTSs [20] and the 3D cell culture model is now considered to be more representative of the tumor architecture and is more often used to study drug activity compared to the 2D model inOvCa [36]. Platelets act as a reservoir of a complex mixture of soluble factors and cellular components, which provide a pro-inflammatory and tumor-promoting microenvironment for tumor cells [3].

Our results demonstrate that PR [27], used as a source of cytokines secreted by activated platelets, increased OvCa-MCTS formation, growth and the expression of the CSC markers ALDH and CD133. Moreover, it counteracted the cytotoxic effects of CDDP, CBDCA and PTX, maintaining high levels of CSC markers after drug treatment.

**Figure 1:**
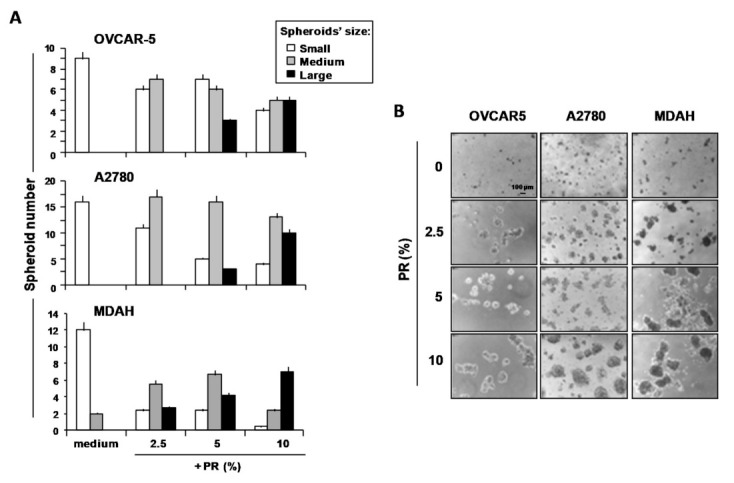
Human platelet releasate (PR) increased spheroid formation in OVCAR5, A2780 and MDAH cells. Cells were cultured on poly-HEMA-coated plates with or without increasing concentrations of PR pools. (**A**) After 5 days, images were captured using an inverted microscope and spheroids were counted (number of cells/spheroid: 2–5 = small; 6–20 = medium; >20 = large). Data are expressed as mean ± SEM of three replicates with three different PR pools. (**B**) Representative phase-contrast photomicrographs (original magnification 4×) showing the formation of ovarian cancer (OvCa) cell multicellular tumor spheroids (MCTSs) in the presence of various PR concentrations.

First, under non-adherent conditions, PR induced the self-aggregation of OvCa cells in MCTSs (Figure 1A,B). Representative phase-contrast photomicrographs of the dose-dependent formation of MTCSs by OVCAR5, A2780 and MDAH treated with PR are shown in Figure 1B.

These results suggest that not only the direct cell contact with platelets [20], but also with molecules released by platelets, may promote the aggregation of OvCa cells as MCTSs in ascitic fluids.

**Figure 2:**
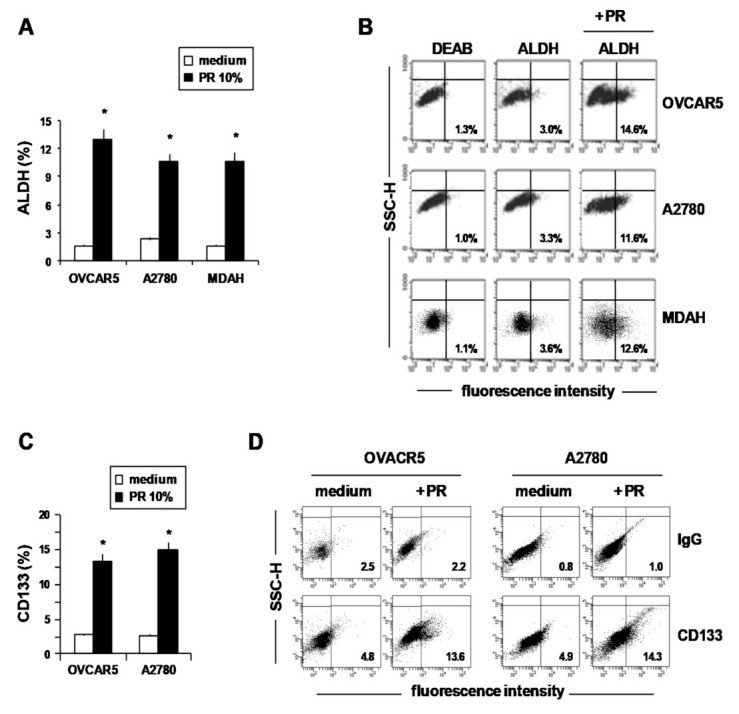
Human PR increased ALDH activity and CD133 expression in OvCa cells. (**A**–**D**) OvCa cells treated for 5 days with or without 10% PR were dissociated into single-cell suspensions by trypsinization, and then ALDH activity and CD133 expression were evaluated by means of flow cytometry. Results represent the percentage of ALDH-positive (**A**) and CD133-positive (**C**) cells. Data are expressed as mean ± SEM of three replicates with three different PR pools. * *p* < 0.01 (untreated vs. PR treated cells). (**B**) Representative flow cytometric dot-plots showing ALDH-positive cells. The ALDH inhibitor diethylaminobenzaldehyde (DEAB) was used as a negative control. (**D**) Representative flow cytometric histograms showing CD133 expression.

Then, we evaluated the CSC markers ALDH and CD133 by means of flow cytometry in OvCa cells cultured with and without PR (10%) (under non-adherent conditions) and found that PR at 10% *v*/*v* significantly increased the percentage of ALDH+ (Figure 2A,B) and CD133+ cells (Figure 2C,D). Representative flow cytometric dot-plots showing ALDH enzymatic activity and CD133 expression by OvCa cells treated or not treated with PR are shown in Figure 2B and Figure 2D, respectively.

These results, showing that PR induced the formation of MCTSs and increased both ALDH and CD133 expression suggest that cytokines secreted by platelets, including TGFβ (Supplemental Figure S1), by favoring the expansion of the drug-resistant cancer stem cells, could decrease the anticancer activity of chemotherapy agents.

At present, the standard treatment for OvCa involves tumor debulking with taxanes, CDDP or CBDCA [37]. These drugs are very effective for OvCa treatment but their toxicity and intrinsic and acquired drug resistance limit their efficacy. To determine the potential protective effects against chemotherapy of PR pre-treatment, we evaluated the cytotoxic effects of CDDP, CBDCA and PTX and the expression of ALDH and CD133 CSC markers in MCTSs obtained through the cultivation of OvCa cells with PR.

Both OVCAR5 and MDAH cells were cultured with PR (10% *v*/*v*) for 5 days (Figure 1A) to allow MTCS formation, then they were treated with CDDP, CBDCA and PTX for 72 h.

**Figure 3:**
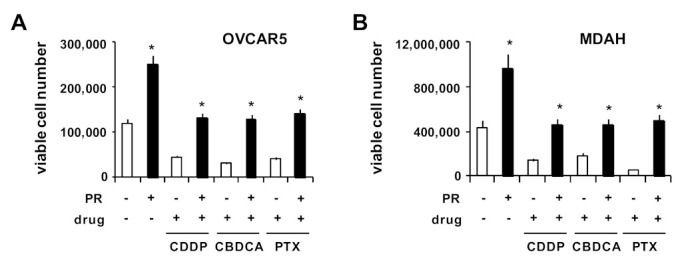
PR increased OvCa cell viability and survival after drug treatment. OvCa cells were cultured for 5 days with PR (10%) on poly-HEMA-coated wells to form spheroids. Then, cells were treated with CDDP, CBDCA and PTX. After 72 h of drug treatment, OvCa spheroids were dissociated into single-cell suspensions by trypsinization and the number of viable cells was evaluated. Histograms showing OVCAR5 (**A**) and MDAH (**B**) viable cell numbers evaluated by the trypan blue dye exclusion assay. * *p* < 0.01 (untreated vs. PR-treated cells).

Treatment of OVCAR5 (Figure 3A) and MDAH (Figure 3B) cells with PR significantly increased the number of viable cells (about 2-fold) and partially counteracted growth inhibition by CDDP, CBDCA and PTX in both cell lines (Figure 3A,B).

**Figure 4:**
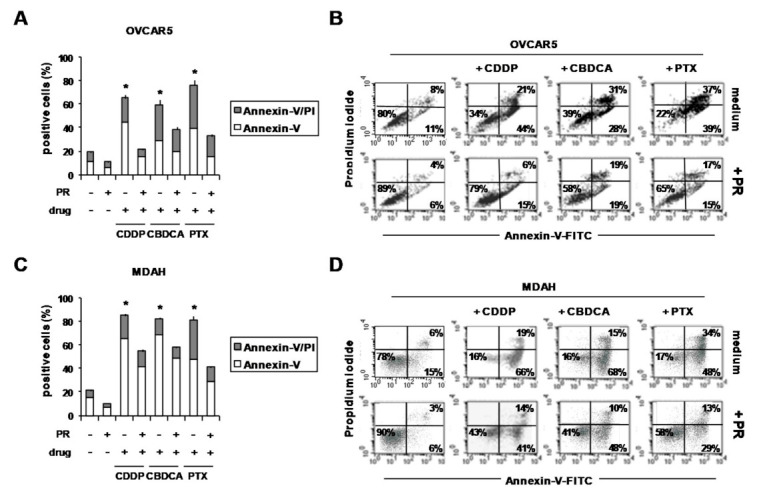
PR reduced the pro-apoptotic effects of CDDP, CBDCA and PTX in PR-OvCa spheroids. OVCAR5 and MDAH cells were cultured for 5 days with PR (10%) in poly-HEMA-coated wells to form spheroids. Then, cells were treated with CDDP, CBDCA and PTX. After 72 h, OvCa spheroids were dissociated into single-cell suspensions by means of trypsinization and stained with Annexin-V-FITC and PI. (**A**,**C**) Histograms showing the percentage of Annexin-V- and Annexin-V/PI-positive cells (**B**,**D**) Representative flow cytometric dot-plots showing the percentage of Annexin-V-, Annexin-V/PI- and PI-positive cells. * *p* < 0.01 (untreated vs. PR-treated cells).

Accordingly, the treatment with PR decreased the pro-apoptotic effects of all drugs (Figure 4). In fact, although drugs increased the percentage of Annexin-V+ (apoptosis) and Annexin-V/PI+ (late apoptosis) in OVCAR5 (Figure 4A,B) and MDAH cells (Figure 4C,D), these effects were partially reduced by PR. Representative flow cytometric dot-plots are shown in Figure 4B,D.

**Figure 5:**
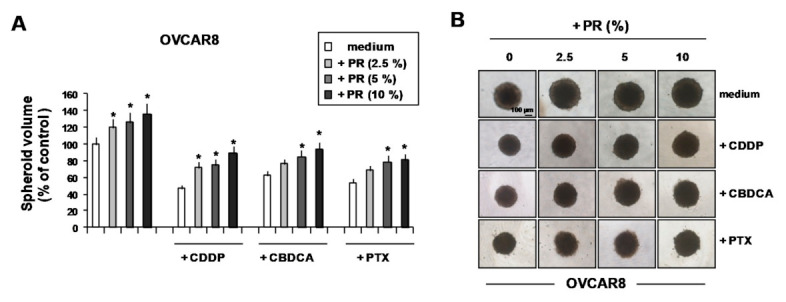
PR increased the volume of OVCAR8 spontaneously-formed single spheroids and decreased their growth inhibition due to CDDP, CBDCA and PTX. Three-day-old OVCAR8 spheroids were treated with increasing concentrations of PR for 24 hours, then for an additional 3 days with CDDP, CBDCA and PTX. (**A**) Responses were evaluated by measuring the volume of at least three spheroids. (**B**) Representative phase-contrast microphotographs showing the volume of OVCA8 spheroids treated with PR, drugs and the combination drugs/PR. Data are expressed as mean ± SD of three replicates with three different PR pools and represent the percentage with respect to control medium. * *p* < 0.05 (untreated vs. PR-treated cells).

PR-mediated cell growth and drug resistance were also evaluated in OVCAR8 cells, which were capable of spontaneously forming compact single MCTSs. As shown in Figure 5A, PR increased OVCAR8 spheroid growth in a dose-dependent manner, as evaluated by spheroid volume, and decreased the growth inhibition induced by CDDP, CBDCA and PTX in a dose-dependent manner (Figure 5A). Representative phase-contrast photomicrographs showing the effects of PR on drug activity in OVCAR8 MCTSs are reported in Figure 5B.

**Figure 6:**
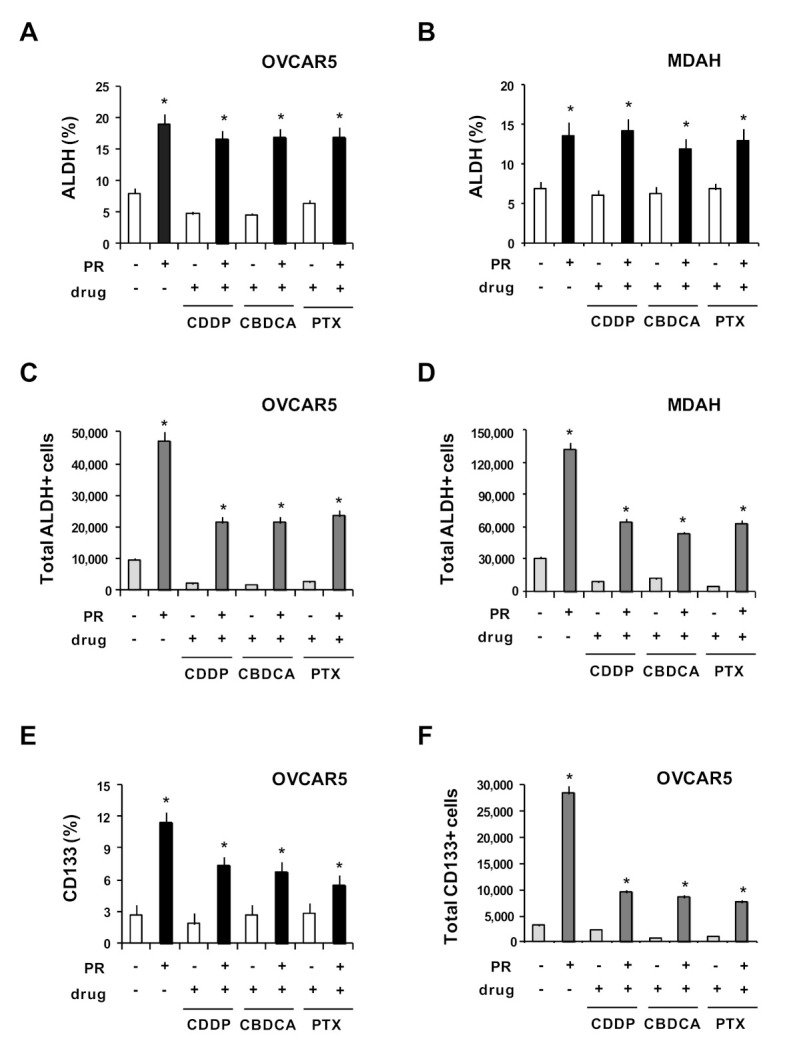
PR increased ALDH- and CD133-positive cells after drug treatment. OVCAR5 and MDAH cells were cultured for 5 days with PR (10%) in non-adherent conditions to form spheroids. Then cells were treated with CDDP, CBDCA and PTX for an additional 3 days. Treated cells were dissociated into single-cell suspensions by means of trypsinization. Histograms show the percentage of ALDH positive cells in OVCAR5 (**A**) and MDAH (**B**). Number of OVCAR5 (**C**) and MDAH (**D**) ALDH-positive cells. Histograms showing the percentage (**E**) and the number (**F**) of OVCAR5 CD133-positive cells. Data are expressed as mean ± SD of three replicates with three different PR pools and represent the percentage with respect to the control medium. * *p* < 0.01 (untreated vs. PR treated cells).

In OvCa, chemotherapy resistance is often associated with high ALDH enzymatic activity [33], a functional regulator [38] and a marker of stemness in many cancer models [39]. ALDH, in combination with CD133, represents a robust marker for OvCa-CSCs [40,41,42]. OvCa-CSCs are thought to contribute to disease recurrence and chemo-resistance; thus, it is challenging to clarify the mechanism leading to their expansion and survival during chemotherapy. We hypothesized that PR could counteract drug activity by allowing the survival of the resistant OvCa-CSCs (ALDH+ and CD133+). We cultured OvCa cells under non-adherent conditions with and without PR for 5 days to generate MCTSs. Then, MCTSs were treated for three more days with a cytotoxic dose of CDDP, CBDCA and PTX ( Table S2). Finally, CD133 expression and ALDH enzymatic activity were evaluated by means of flow cytometry. PR increased the percentage of ALDH+ cells, which was maintained in OVCAR5 (Figure 6A) and MDAH cells (Figure 6B) after treatment with drugs and PR. PR, by increasing the number of viable cells, together with the percentage of ALDH+ cells, also strongly increased the total number of ALDH+ cells in drug-treated OVCAR5 (Figure 6C) and MDAH cells (Figure 6D), with respect to untreated cells. Indeed, ALDH^high^ OvCa CSCs display an enhanced ability to form spheroids, generate metastasis and resist apoptosis and drug activity [35,43]. ALDH^high^ OvCa CSCs are enriched in residual xenografts after platinum therapy and are 100-fold higher in OvCa cells selected for taxane-resistance in vitro [43]. Accordingly, ALDH expression increases in residual tumors after the first round of chemotherapy, compared to tumors from untreated patients [43].

The percentage of CD133+ cells increased in the presence of PR, but was reduced after drug treatment (Figure 6E); nevertheless, the total number of CD133+ cells remained significantly higher with respect to OvCa cells without PR (Figure 6F). In line with preclinical and clinical studies demonstrating that in cancer patients and mouse models of OvCa high platelet counts were related to a poor responses to chemotherapy (platinating agents and taxanes) [5,44], our results suggest that platelet products, by promoting the expansion of CD133 and especially ALDH+ cells (the putative OvCa-CSCs), could promote their survival during chemotherapy.

PR contains several growth factors that have been involved in OvCa progression, including CSCs expansion and drug resistance (Supplemental Table S2)—EGF, secreted by M2-like TAMs, activates the EGFR-ERK signaling pathway and promotes the progression of OvCa [45,46]; EGFR blockade targets ALDH-CSCs and reverses cisplatin resistance [47,48,49]; anti-IGF-1R-targeted strategies potentiate the efficacy of platinum-based chemotherapy [50,51]; PDGF, secreted by mesenchymal stromal cells (MSCs), induces platinum resistance and CSC enrichment (CD133, and ALDH in OvCa heterospheroids) [52]; TGFβ increases OvCa growth [18,22,53] and its neutralization potentiates tumor immunity and decreases OvCa progression [54,55]; CCL5 and CCR5 are mainly expressed by CD133+ OvCa stem-like cells [56], CCL2/CCL5, secreted by stromal cells, induces IL-6/PYK2-dependent chemoresistance in OvCa cells [57] and cisplatin, by inducing the secretion of CCL5 by cancer-associated fibroblasts (CAFs), promotes cisplatin resistance [16]. Thus, the interaction of OvCa cells with platelets [20] and their products may contribute to the expansion of ovarian CSCs, as well as to decreased drug activity and consequently to tumor progression [58].

## 3. Materials and Methods

### 3.1. Drugs

Cisplatin (CDDP), purchased from Mayne Pharma (Naples, Italy), carboplatin (CBDCA) from Teva Pharmaceutical Industries (Milan, Italy, S.r.l), and Paclitaxel (PTX, TAXOL^®^) from Accord Healthcare Italia (Milan, Italy) were surplus drugs from the clinical pharmacy of CRO Aviano.

### 3.2. Platelet-Releasate (PR) Production and Cytokine Evaluation

Pools of PR were obtained as previously described [27]. Briefly, leukocyte-depleted PR was obtained by plasma platelet apheresis from healthy donors. Platelet activation was performed in PR via the addition of CaCl_2_. Three separate samples were pooled together to obtain 3 different pools that were used to perform experiments. Concentrations of PDGF-AA, PDGF-AB, PDGF-BB, TGF-β, FGF, EGF, IGF-1, VEGF and CCL5 were measured in the 3 different pools (Supplemental Table S1), using commercially available Quantikine ELISA kits (R&D Systems, Minneapolis, MN, USA) according to the manufacturer’s instructions.

### 3.3. Cell Lines and Culture Conditions

The A2780 cell line (endometrioid adenocarcinoma) was from Sigma (Inc., St. Louis, MO, USA); MDAH-2774 (CRL-10303) (high grade endometrioid-carcinomas) was from ATCC. Dr. Baldassarre (CRO, Aviano, Italy) provided OVCAR5 and OVCAR8 (high grade ovarian serous adenocarcinoma) cells. All cell lines were routinely tested for mycoplasma, with negative results, and authenticated in our laboratory using a PowerPlex 16 HS System (Promega, Madison, WI, USA) and GeneMapper ID version 3.2.1 to identify DNA short tandem repeats. Cells were cultured in AQ MEDIA-RPMI-1640 (R2405, Sigma-Aldrich-Italy) supplemented with 10% heat-inactivated fetal bovine serum (FBS, F2442FBS; Sigma), 0.2 mg/mL penicillin/streptomycin (P4333, Sigma) and 0.1% (*w*/*v*) L-glutamine (Sigma) at 37 °C in a 5% CO_2_ fully humidified atmosphere.

### 3.4. Spheroid Formation, Growth and Treatment

To generate spheroids, cells were cultured under non-adherent conditions in 12-well plates coated twice with 20 mg/mL of poly(2-hydroxyethyl methacrylate) (poly-HEMA; Sigma, P3932) in 95% ethanol and washed once with PBS before cell seeding. Cells (10 × 10^3^/mL) were cultured in RPMI medium plus 1% FCS and treated with increasing concentrations of PR pools (2.5%, 5% and 10%). After 5 days, spheroids were counted and captured using an inverted microscope (Eclipse TS/100, Nikon) with a DS Camera Control Unit DS-L2 photomicrographic system. To evaluate drugs’ effects, spheroids were treated with CDDP, CBDCA and PTX for 3 additional days. Then, spheroids were dissociated into single-cell suspensions by trypsinization for further analysis [25]. The half maximal inhibitory concentrations (IC_50_) for each drug, previously calculated in OvCa cell layers, are shown in Supplemental Table S2. Drug activity was also evaluated on preformed single spheroids, as previously described [26]. Briefly, OVCAR8 cells (500 cells/well) were seeded in a 96-well round-bottom plate coated with poly-HEMA (Sigma) to generate single spheroids with a defined size. After 3 days, OVCAR8 spheroids were treated with increasing concentrations of PR for 24 hours, then for an additional 3 days with CDDP, CBDCA and PTX. Drug activity was evaluated through the measurement of spheroid size [26] using an inverted microscope (Eclipse TS/100, Nikon) and spheroids’ volumes were calculated using the formula width^2^ × length × 3.14)/6 [59].

### 3.5. Flow Cytometry

Spheroids were dissociated into single-cell suspensions by means of trypsinization. Then, Annexin-V-FITC (556419, Becton Dickinson Pharmingen, Milano, Italy) binding, CD133 expression (AC133-PE, 130-080-801, Miltenyi Biotec S.r.l. Bologna, Italy) and aldehyde dehydrogenase (ALDH) enzymatic activity (Aldeflour, 01700) (Stem Cell Technologies, Inc., Cambridge, MA, USA) were evaluated as previously described [25]. Viable ALDH+ labeled cells were identified according to their forward and right-angle scattering, electronically gated and analyzed on a FACSCalibur flow cytometer (BD), using CellQuest Software (BD). The total number of ALDH+ cells was calculated by multiplying the percentage of ALDH-positive cells by the recovered OvCa cells after drug and PR treatment. TGFβ1-blocking antibody (TB21) (GTX21279) (GeneTex Inc.) (Prodotti Gianni, Milano, Italy) was used.

### 3.6. Statistics

Statistical analysis was performed using GraphPad Prism 6 Software (GraphPad). The significance of differences was determined by Student’s *t*-test for comparisons between two groups. Analysis of variance was used to evaluate the correlation of data among three or more groups; consecutive multiple comparison analysis was performed using Dunnett tests. * *p* < 0.05 was considered statistically significant.

## 4. Conclusions 

Platelets and their products, by maintaining high levels of CSCs during drug treatment and favoring the expansion of drug resistant clones, may determine the failure of therapy and even induce relapse with a drug-resistant disease and may be considered new actors in tumor progression, mediated by the tumor microenvironment. In this context, targeting platelets with anti-platelet drugs [21], such as aspirin [60], or receptors activated by platelet-secreted growth factors [47] may expand the therapeutic tools available to improve standard chemotherapy. Finally, PR may help to define the molecules involved in CSC expansion and drug resistance and to find new therapeutic targets in OvCa.

## Authors Contributions

Conceptualization, methodology and validation, N.C., C.B. and D.A.; investigation, formal analysis, N.C., C.B., C.D. and F.A.; data curation, N.C., C.B. and F.A.; writing—original draft preparation D.A.; writing—review and editing, D.A., N.C., A.C. and M.M.; supervision, D.A.; funding acquisition D.A. and M.M. All authors have read and agreed to the published version of the manuscript.

## Data Availability

Data is contained within the article or Supplementary Material.

## Supplementary Materials

The following are available online at https://www.mdpi.com/1422-0067/22/6/3019/s1. Table S1: Concentrations of growth factors in platelet releasate; Table S2: Half-maximal inhibitory concentrations (IC50) of carboplatin (CDBCA), cisplatin (CDDP) and paclitaxel (PTX) in ovarian cancer cell lines OVCAR5, OVCAR8 and MDAH; Figure S1: Anti-TGFβ inhibited the increase of ALDH activity and CD133 expression by PR in OvCa cells.
