# Supplementary material for: In Ovarian Cancer Multicellular Spheroids, Platelet Releasate Promotes Growth, Expansion of ALDH+ and CD133+ Cancer Stem Cells, and Protection against the Cytotoxic Effects of Cisplatin, Carboplatin and Paclitaxel
Source: Int J Mol Sci. 2021 Mar 16;22(6):3019. doi: 10.3390/ijms22063019 (PMC7999151; doi:10.3390/ijms22063019)
Supplement: Supplementary file 1 [file ijms-22-03019-s001.pdf]

Supplemental Table S1. Concentrations (pg/ml) of growth factors in PR pools.

	pool 1	*pool 2*	*pool 3*
EGF	978	971	1091
VEGF	844	739	1030
FGF	11	8	9
IGF-1	79074	72500	83422
PDGF-AA	8948	11777	10201
PDGF-AB	52940	60230	44794
PDGF-BB	22103	22361	20148
TGF-β	83900	81068	84401
CCL5	635	771	698

Growth factor concentration values evaluated by Elisa assay measured in 3 different PR pools.
EGF, epidermal growth factor; VEGF, vascular endothelial growth factor; FGF, fibroblast growth factor; IGF-1, insulin-like growth factor-1; PDGF-AA, -AB and -BB, platelet-derived growth factor isoforms AA, AB and BB; TGF-β, transforming growth factor beta; CCL5, chemokine (C-C motif) ligand 5.

Supplemental Table S2. Growth inhibition by carboplatin, cisplatin and paclitaxel in OVCAR5, OVCAR8 and MDAH ovarian cancer cell lines.

	OVCAR5		OVCAR8		MDAH	
drug	IC_{50}	IC_{75}	IC_{50}	IC_{75}	IC_{50}	IC_{75}
CDBCA (μM)	89.2 ± 8.0	123.2 ± 11.1	59.8 ± 0.5	124.6 ± 11.2	21 ± 1.9	83 ± 7.5
CDDP (μM)	11.9 ± 1.0	40.1 ± 3.6	6.1 ± 0.4	17.8 ± 0.8	4.3 ± 0.4	10.2 ± 0.9
PTX (ng/ml)	25.3 ± 2.2	69.2 ± 6.2	155.3 ± 16.9	489.5 ± 30.3	2.6 ± 0.3	3.4 ± 0.2

Cell lines were exposed to increasing concentrations of carboplatin (CDBCA), cisplatin (CDDP) and paclitaxel (PTX). After 72 h viable cells were evaluated by MTT assay. Results represent the mean ± SD of three replicate wells from three independent experiments. IC_{50} and IC_{75} values were calculated using the Calcusyn software (1).

(1) Chou TC, Talalay P. Quantitative analysis of dose-effect relationships: the combined effects of multiple drugs or enzyme inhibitors. Adv Enzyme Regul 1984;22:27-55.

Supplemental Figure S1

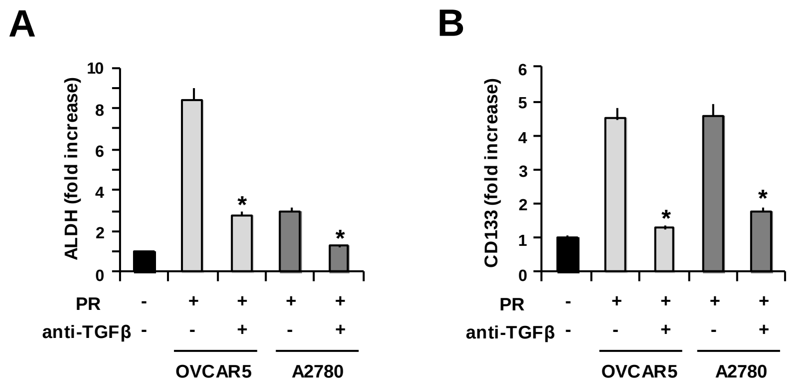

Supplemental Figure S1. Anti-TGFβ inhibited the increase of both ALDH activity and CD133 expression by PR in OvCa cells. OVCAR5 and A2780 cells were treated for 5 days with or without 10% PR and TGFβ blocking antibody (5 μg/ml)[1]. Then cells were dissociated into single-cell suspensions by trypsinization and ALDH activity and CD133 expression were evaluated by flow cytometry. Results represent the percentage of ALDH (A) and CD133 (B) positive cells and are expressed as fold increase respect to untreated cells. **P*<0.01 (PR treated vs. anti-TGFβ-PR treated cells).

1. Cho MS, Bottsford-Miller J, Vasquez HG, Stone R, Zand B, Kroll MH, et al. Platelets increase the proliferation of ovarian cancer cells. Blood. 2012;120:4869–72.
